# Growth of Bone

**Published:** 1864-06

**Authors:** 


Growth of Bone.—M. De Lamballe appears, in the course of his extensive researches on the generation and reparation of tissues, to have corroborated the assertions of M. Flourens. 1. That the bones increase in thickness by external and superimposed layers. 2. That they increase in length by the addition of terminal layers arranged in juxtaposition. 3. That proportionally as the new layers are deposited externally, the older ones on the inner surface are resorbed. 4. That ossification consists in the regular and successive conversion of periosteum into cartilage, and of cartilage into bone.—*Popular Science Review.*

